# Cigarette craving in virtual reality cue exposure in abstainers and relapsed smokers

**DOI:** 10.1038/s41598-024-58168-7

**Published:** 2024-03-30

**Authors:** Benedikt Schröder, Agnes Kroczek, Leon O. H. Kroczek, Ann-Christine Ehlis, Anil Batra, Andreas Mühlberger

**Affiliations:** 1 Department for Psychology, Clinical Psychology and Psychotherapy, University of Regensburg, Universitätsstraße 31, 93053 Regensburg, Germany; 2 Department of Psychiatry and Psychotherapy, Tübingen Center for Mental Health (TüCMH), University Hospital Tübingen, Tübingen, Germany; 3 Department of Psychiatry and Psychotherapy, Tübingen Center for Mental Health (TüCMH), Section for Addiction Research and Medicine University Hospital Tübingen, Tübingen, Germany; 4 German Center for Mental Health (DZPG), partner site Tübingen, Tübingen, Germany

**Keywords:** Psychology, Health care

## Abstract

Cue exposure therapy (CET) in substance-use disorders aims to reduce craving and ultimately relapse rates. Applying CET in virtual reality (VR) was proposed to increase its efficacy, as VR enables the presentation of social and environmental cues along with substance-related stimuli. However, limited success has been reported so far when applying VR-CET for smoking cessation. Understanding if effects of VR-CET differ between future abstainers and relapsing smokers may help to improve VR-CET. Data from 102 participants allocated to the intervention arm (VR-CET) of a recent RCT comparing VR-CET to relaxation in the context of smoking cessation was analyzed with respect to tolerability, presence, and craving during VR-CET. Cue exposure was conducted in four VR contexts (Loneliness/Rumination, Party, Stress, Café), each presented twice. Relapsed smokers compared to abstainers experienced higher craving during VR-CET and stronger craving responses especially during the Stress scenario. Furthermore, lower mean craving during VR-CET positively predicted abstinence at 6-month follow-up. Attempts to improve smoking cessation outcomes of VR-CET should aim to identify smokers who are more at risk of relapse based on high craving levels during VR-CET. Specifically measuring craving responses during social stress seems to be well suited to mark relapse. We propose to investigate individualized treatment approaches accordingly.

## Introduction

Craving is a diagnostic criterion for substance-use disorders according to DSM-5 and is defined as longing or urge for the substance, which can occur at any time but is more likely in an environment where the substance was previously obtained or consumed^1^. Most theoretical models of addiction consider craving as a central motivational component of ongoing substance use and closely linked to relapse^2^. Among smokers, craving is typically increased in the presence of smoking-related stimuli^3^, rises in phases of abstinence^4^, and is reported as the most distressing symptom of a smoking cessation attempt^2^. However, as reviewed for several substance-use disorders^5^ and also specifically with respect to smoking^6^, there are inconsistent results as to whether craving is able to predict relapse. Tiffany et al.^2^ (p. 181) suggest that conflicting findings might be explained partly by the fact that the association of craving with maintained substance use and relapse only exists when “the conditions of craving assessment and/or induction are maximally representative of the natural expression of craving.” However, this representativeness is limited in clinical settings by the fact that they do not provide a typical context of consumption.

Cue reactivity (CR) is a motivational response to drug-related cues and is considered a core characteristic of addiction as drug consumption is strongly influenced by cues (e.g. environments, objects, emotions) previously associated with the drug's effects through classical conditioning^7^. CR consists of three modalities: a physiological reaction, a behavioral reaction (e.g. drug-seeking), and craving^8^. Of these, self-reported craving is most commonly used as a measure of CR and was found to be a stronger indicator of CR than physiological variables^7^.

Drawing from the successful treatment of anxiety disorders through exposure^9^, cue exposure therapy (CET) has been proposed in the treatment of substance use disorders to reduce CR and ultimately the probability of relapse since both disorders have in common that conditioning processes were proposed to be responsible for their maintenance and to be targeted during treatment^10^.

CET aims to reduce the conditioned response (i.e. craving) to conditioned stimuli (e.g. smoking paraphernalia) through extinction learning, which is supposed to be achieved by repeated confrontation with the conditioned stimuli without smoking^9^. Despite this plausible treatment rationale, CET has not yet proven to be a consistently effective intervention in the treatment of substance use disorders^11–13^. A possible cause for this could be that traditional CET in substance use disorders usually presents substance-related stimuli in a clinical setting in the form of images, videos, or tactile objects such as wine glasses or bongs, which is accompanied by relatively low ecological validity^14^. It has been argued that this limitation might be overcome by applying CET in virtual reality (VR-CET) because in addition to substance-associated objects, environments and social contexts, including interactions (e.g. consumption offers) and emotional triggers, can be presented that more closely reflect real-life situations^15^, thus promoting both cue and contextual extinction^9,14^. This is important as craving usually increases due to a complex combination of environmental cues, emotional states, reactivation of autobiographical experiences, and sensory information^16^. Accordingly, VR cue exposure reliably induced craving across various substance use disorders including nicotine^17^.

Early treatment studies supported the feasibility of VR-CET in smoking cessation and found concomitant decreases in craving^18^ and lower smoking rates and craving in smokers receiving VR-CET and nicotine replacement therapy compared to nicotine replacement therapy alone^19^. In addition, a preliminary study with a small sample size compared cognitive behavioral therapy (CBT) alone with VR-CET alone and found both treatments comparably effective^20^. Another study, though without a control group, reported that VR-CET reduced the number of smoked cigarettes per day, carbon monoxide in exhaled air, and craving across exposure sessions^21^. Surprisingly, however, a recent RCT with a long follow-up period found that CBT combined with VR-CET was not superior to CBT alone in terms of abstinence rates and thus could not demonstrate specific efficacy of VR-CET^22^. A question important for understanding the effect of VR-CET that has so far remained unaddressed in the aforementioned VR-CET studies, is whether individuals who later achieve abstinence differ in terms of their craving from those who do not. In addition, there is a lack of research examining potentially relevant aspects of VR-CET aside from craving such as emotion induction, self-efficacy, presence, and tolerability.

The goal of the present study was to identify possible differences in the course of craving during VR-CET in future successful abstainers compared to relapsing smokers in order to obtain indications for future improvements of VR-CET, and to clarify the predictive ability of craving in ecologically valid contexts. In addition, we aimed to evaluate the scenarios used for VR-CET in terms of emotion induction, presence, and tolerability.

We hypothesize that smokers who relapse within 6 months after completing treatment in principle experience stronger craving in VR-CET than those who maintain abstinence. We additionally hypothesize that craving during VR-CET is predictive for future medium-term relapse.

## Methods

### Design and participants

This study analyzes data from participants allocated to the intervention arm of a bicentric RCT (ClinicalTrials.gov Identifier: NCT03707106), which investigated the efficacy of cue exposure therapy in virtual reality (VR-CET) in the context of smoking cessation. In this RCT, VR-CET was compared to an unspecific relaxation intervention (control group). Both arms were incorporated into an already evaluated cognitive-behavioral smoking cessation group therapy. The study protocol^23^ and the primary results are reported elsewhere. All participants provided written informed consent, and the RCT was approved by the Ethics Committee of the Faculty of Medicine at the University Hospital of Tuebingen (no. 836/2016BO1) and by the Ethics Committee of the German Psychological Society (DGPs) (no. AM022017). The study was conducted according to the approved procedures. Inclusion criteria were being between 18 and 70 years of age and smoking a minimum of 10 cigarettes daily for at least 2 years. Exclusion criteria were pregnancy, participation in another smoking cessation program within 6 months before assignment, current diagnosis of a psychiatric disease including current depression or substance use disorder (other than nicotine), and lifetime diagnosis of psychosis, bipolar affective disorder or posttraumatic stress disorder.

The intervention arm included a total of 122 randomized participants of which 102 participants received the allocated VR-CET (n = 20 dropped out before the start of the intervention). As the present study focuses on the processes during VR-CET, the current sample consists of the 102 participants who received VR-CET, including 43 female (42.2%) and 59 male smokers (57.8%) aged M = 45.67 years (SD = 13.40; range 20–68 years). On average participants attended three out of a total of four VR-CET appointments (M = 3.00, SD = 1.03). Prior to starting smoking cessation, participants in the analyzed sample reported to smoke M = 17.93 cigarettes per day (SD = 5.81) and they scored at M = 4.46 points (SD = 2.04) on the Fagerström Test for Nicotine Dependence^24^ (FTND). The mean age at smoking initiation was 17.44 years (SD = 4.78). The exhalation carbon monoxide (CO) level at the beginning of smoking cessation was M = 17.23 (SD = 11.09). Among this sample, 24 participants (23.5%) remained continuously abstinent 6 months after treatment while 78 participants (76.5%) relapsed (intention to treat). Prior to the smoking cessation intervention, abstainers and relapsed smokers did not differ significantly in terms of cigarettes per day (*p* = 0.739), FTND scores (*p* = 0.169), age of regular smoking onset (*p* = 0.713), age at time of study inclusion (*p* = 0.849), and CO level at the first appointment of smoking cessation (*p* = 0.647). In addition, abstainers and relapsed smokers did not differ significantly regarding gender (*p* = 0.317).

During treatment, in the days before the first VR-CET session, 81 participants were abstinent (validated by results of < 10 ppm in CO measurement) and 16 participants still smoked. In the days before the second VR-CET session, 71 participants were abstinent and 5 participants smoked. In the days before the third VR-CET session, 64 participants were abstinent and 8 participants smoked. In the days before the fourth VR-CET session, 61 participants were abstinent and 9 participants smoked.

### Apparatus and VR scenarios

VR was presented with the VIVE Pro head-mounted display (HMD; HTC Corporation, Taoyuan, Taiwan). Integrated headphones played the sound. Hardware to run the VR included the NVIDIA GeForce GTX 1080 Ti graphics card (Nvidia Corporation, Santa Clara, CA, United States), the Intel(R) Core(TM) i7-7740X CPU @ 4.30 GHz (Intel Corporation, Santa Clara, CA, United States), 16 GB DDR4 RAM, and the Samsung SSD 850 hard disc (Samsung Group, Suwon, South Korea). The VR environment was controlled using CyberSession Research 5.8 (VTplus GmbH, Würzburg, Germany). Participants used a gamepad (F710 Wireless Gamepad, Logitech international S.A., Apples, Switzerland) to navigate within the VR. Experimenters were able to follow participants' view and audio via a separate computer screen and speaker (set to the lowest volume and turned in the direction of the investigators away from the participants to avoid double hearing of the audio for participants).

A total of four virtual environments were presented, each lasting between approx. 13 and 18 min. All environments included standardized pre-recorded instructions that were played via the headphones. Two of the VR environments described below (Loneliness/Rumination and Stress) were created using the Steam Source engine (Valve Corporation, Bellevue, WA, United States) at the Department of Psychology (Clinical Psychology and Psychotherapy) at the University of Regensburg. Another two VR environments (Party and Café) were created with Unreal Engine 4 (Epic Games, Raleigh, NC, United States) by VTplus GmbH (Würzburg, Germany).

Each VR environment contained three-dimensional smoking-related stimuli (e.g. cigarette packs, loose cigarettes, lighters, ashtrays). The cigarette packets were individually configured to the participants' preferred smoking brand from a list of 21 tobacco brands. If the own brand was not included, the preferred brand among the existing brands was selected. The RCT did not assess how frequently this occurred. Inquiries among investigators indicated that the participants' own brand was mostly available for selection in VR, which is supported by the fact that the brands available for selection included the 9 largest cigarette brands in Germany in terms of market share^25^.

**Figure 1:**
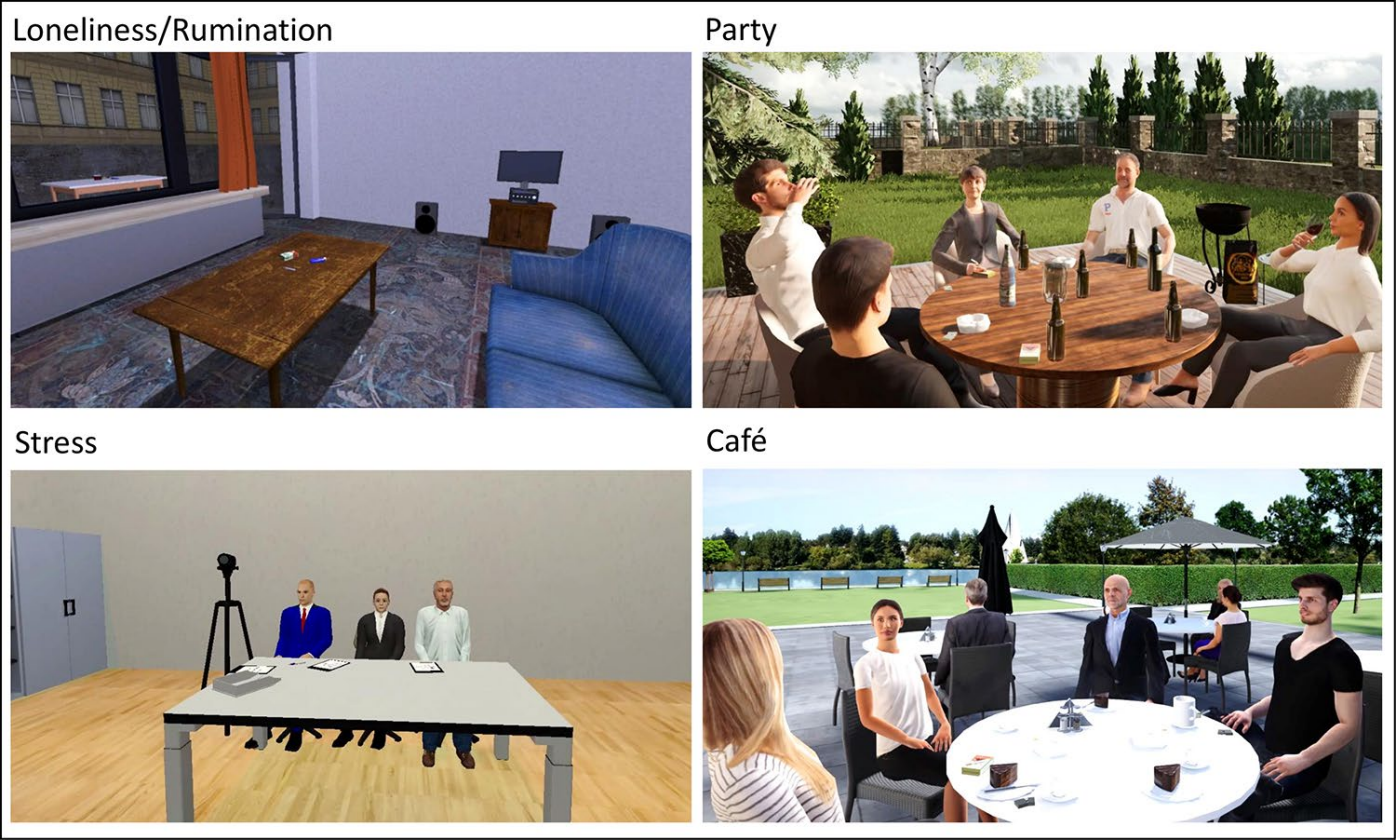
Screenshots of the virtual environments used for cue exposure. Persons depicted are virtual agents and not real persons.

Development of VR environments was based on a survey of participants in a smoking cessation course^23^. In the following we describe the four VR scenarios used for cue exposure in detail (see Fig. 1 for screenshots of the scenarios). (1) Loneliness/Rumination: Before the VR session, investigator and participant select the most burdensome concern from the participant's daily life based on results of the German version of the Worry Domains Questionnaire^26^ (WDQ-D) already completed at baseline. After putting on the HMD, the participants are instructed to explore the VR environment consisting of a living room and a balcony freely for one minute using the gamepad. Participants are then assisted in sitting down and are instructed to describe the selected concern and the associated mood, thoughts, feelings, and physical experiences. They are further instructed to ruminate on the selected concern (worry induction). Participants sit alone on a sofa while smoking paraphernalia (cigarette pack, lighter, six loose cigarettes) are displayed on a table in front of them. In the direction of the participants' view, there is a window facade with an open door to a roofed balcony. On a table on the balcony an ashtray is placed and there is another pack of cigarettes and a lighter. Rain is visible and audible through the windows. Slow piano music comes from a stereo. Following worry induction, participants are instructed to inspect the smoking paraphernalia in detail (specifically to perceive the color of the lighter, the color and the logo of the cigarette pack, and to count the number of cigarettes). The scenario lasts approx. 18 min. (2) Party: Participants are immersed in a virtual barbecue party and are informed that they will receive cigarette offers and that their task is to firmly refuse them. Initially, participants are given one minute to freely navigate within the VR environment. After that, the participants are assisted to sit down. In VR, participants are placed at a table together with five virtual agents (two female, three male), three of whom are currently smoking. Two other characters also light a cigarette in the course of the scenario. Three characters drink beer, one red wine, and one water. There are cigarette packs, lighters, and ashtrays on the table. The terrace is surrounded by a garden, a barbecue is next to the table, and the sun is shining. The smokers talk about the pleasure of smoking and how well smoking fits the current situation. One character offers the only character not yet smoking a cigarette, which the character refuses on the grounds that he has recently quit smoking. One smoking character replies that he had already tried to quit, but wonders why he should put himself through this strain when he enjoyed smoking so much. While the four smoking characters continue to talk about the pleasurable aspects of smoking, the previously declining character changes his mind saying he wants a cigarette after all and starts to smoke as well. Over the course of this scenario, participants receive four offers of cigarettes, at about 3.5, 6, 6.5 and 7 min after the start of the scenario. Overall, the scenario lasts approx. 13 min. (3) Stress: This scenario is based on the Trier Social Stress Test paradigm^27^ (TSST), which has already been able to generally induce psychosocial stress in previous VR adaptations^28^ and also specifically in smokers^29^. In the present adaptation, participants begin this scenario in a standing position in an office space. In the room there is a desk and several chairs. One end of the room consists of a glass front with an open door to a terrace surrounded by trees. On the terrace the sun is shining and one character is smoking next to a floor-standing ashtray. Additionally, there is a closed opaque door next to which hangs a sign that states "Interviews". Participants are again informed that they should decline cigarette offers during the course of the scenario. They are further informed that they participate in an interview for their dream job and should now prepare to give a talk about their own strengths and their particular suitability for the job. It is announced that this talk would subsequently be presented to an application committee of three psychologists trained in behavioral observation. The participants are also informed that there are three other applicants, one of whom is currently in the interview room, another is smoking on the terrace, and one has not yet arrived. After two minutes of preparation time, the third applicant enters the room, mentions how nervous she is, notices the character smoking on the terrace and states that she now also needs a cigarette. Before going to the terrace, she also offers a cigarette to the participant. Once the offer is declined, she answers that she has enough cigarettes in case the participant wants one later and starts smoking next to the character already standing on the terrace. Next, a character from the interview room approaches the participant and informs that the preparation time is over and that she is now accompanying the participant to the committee. Participants are then led into the interview room and are asked to stand in front of a committee (three persons) and a camera. Subsequently, participants perform their presentation for two minutes while standing in front of the expressionless committee. If participants finish their presentation before the end of the two minutes, one committee member asks to continue the presentation stating there is still time available (triggered by the investigator). Upon completing the presentation, a previously unannounced arithmetic task is introduced by the committee. Participants are instructed to continuously subtract 17 from 2023 and to calculate as quickly and correctly as possible. The arithmetic task lasts two minutes and participants are prompted by the committee to start over at 2023 if they made a mistake. After these tasks, participants find themselves together with the two smoking competitors on the terrace. The competitors talk about the relaxing and rewarding effect of smoking during this stressful situation and one competitor offers the participant a cigarette a total of three times, at about 4, 10 and 10.5 min after the start of the scenario. Overall, the scenario lasts approx. 17 min. (4) Café: Participants begin this scenario standing on a terrace of a café and are informed that they will receive cigarette offers which they should refuse firmly. Participants are given one minute to freely navigate within the VR environment. Next, the participants are assisted to sit down at a table of the café. Four characters (two female, two male) are already sitting at the table. On the table there are plates with cakes, cups, two ashtrays, two cigarette packs and lighters. The weather is good. A park and a lake are visible in the background. The characters first talk about the beautiful weather, about the nice atmosphere at the lake, and about the delicious cake and coffee. They further talk about how well-fitting cigarettes and coffee are and the four characters start smoking. One character offers the participant a cigarette. After the participant's refusal, the characters express their opinion that one should not forbid oneself every pleasure in life and that smoking is part of the enjoyment at that particular moment. More conversation follows about a previous overnight stay together at a mountain hut, where they had always gone out together to smoke. In addition, they talk about earlier bar visits when it was still allowed to smoke in bars and how pleasant those times were without smoking restrictions. They state, that for them, smoking is associated with freedom, which they do not want to be taken away. During the conversation described above, the first offer of cigarettes is followed by an additional two offers. The offers occur at about 3, 4.75 and 9.25 min after the start of the scenario. Overall, the scenario lasts approx. 16 min.

### Procedure

**Figure 2:**
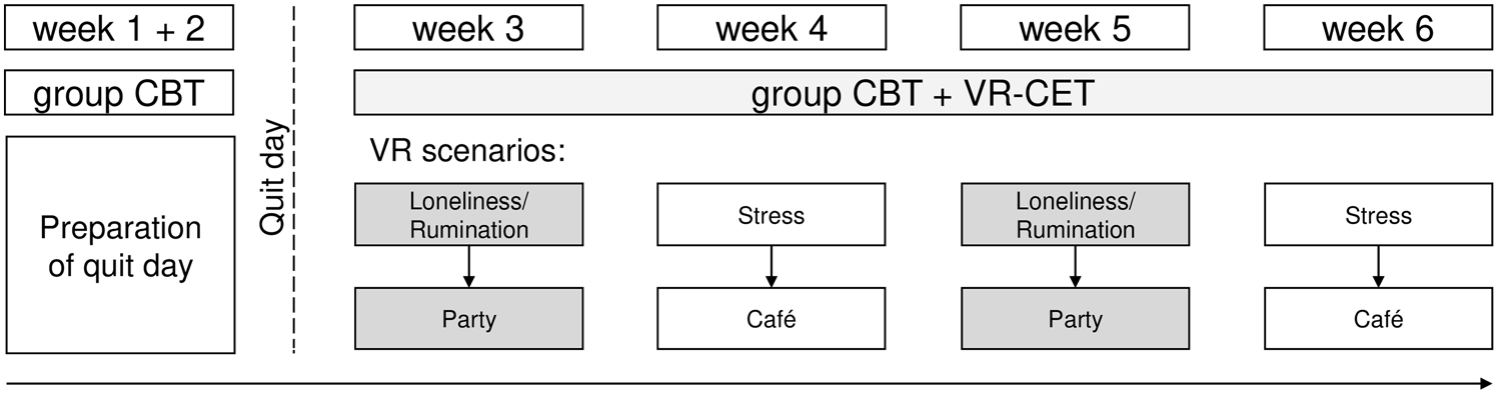
Sequence of treatment weeks and VR-CET sessions.

This description focuses on the VR-CET intervention arm (see Fig. 2 for a schematic overview). The overall procedure of the RCT can be found elsewhere^23^. After random allocation to VR-CET, a 6-week group-based cognitive behavioral smoking cessation program^30,31^ started in group sizes of 6 to 8 participants and with weekly appointments. Each group session lasted 90 min and was scheduled for late afternoon or evening. In session 2, the treatment rationale for CET was introduced. In addition, session 2 involved scheduling a date to quit smoking in the upcoming week prior to session 3. VR-CET took place individually in sessions 3 to 6 and lasted an additional 40 min per session. With two VR systems per study center, part of the participants completed the VR-CET before the group session and part of them afterwards. VR-CET took place in a separated room for each participant. The investigator being in the same room as the participant repeated the CET rationale, assisted in putting on the HMD, informed about steering in VR, selected the tobacco brand, noted the participants' answers to rating questions, and controlled the VR. Controlling the VR involved starting and stopping of the VR session as well as reacting to the participants' voice by continuing to the next part of the VR scenario by button press whenever interactions between the participants and the VR such as rejecting cigarette offers took place. Each VR-CET session included the successive completion of two scenarios. VR-CET started with the scenarios Loneliness/Rumination and Party, followed by Stress and Café in session 4. Session 5 was identical to session 3, while session 6 was identical to session 4. After each VR session, participants completed the VR-related questionnaires.

### Measures

Measures of the RCT included in the present analysis consist of questionnaires, rating questions, and the clinical outcome measure.

*Questionnaires:* At study inclusion, participants completed a demographic questionnaire, a smoking questionnaire documenting smoking history and current smoking behavior, the Fagerström Test for Nicotine Dependence^24^ (FTND), and the German version of the Worry Domains Questionnaire^26^ (WDQ-D). After each session of VR-CET, the experience of presence during VR was measured with the Igroup Presence Questionnaire^32^ (IPQ). The potential occurrence of cybersickness symptoms (i.e. headache, nausea, vertigo) was assessed with a brief version of the Simulator Sickness Questionnaire^33^ (SSQ-b).

*Ratings:* All rating questions should be answered by the participants with a number between 0 (lowest rating) and 100 (highest rating). To check whether the scenarios induced the target emotions (worry in Loneliness/Rumination, stress in Stress, and sociability in Party and Café), the intensity of each emotion was rated at the end of each scenario using the following questions: “How worried were you in the situation?”, “How stressed were you in the situation?”, and “How much sociability did you feel in the situation?”. Craving ratings (4 to 6 ratings depending on the scenario) were presented before, during (e.g. after having received cigarette offers) and after each session of VR-CET with the question “How strong is your current desire to smoke a cigarette?”. At the end of each VR scenario, an additional craving rating phase existed with a total of 8 craving ratings conducted at 30-s intervals during which the VR environment continued to be presented. If the study participants' answer was 0 two times in a row, the remaining questions of the craving rating phase were skipped. For the analysis, the remaining values following a two-time response of 0 were set to 0. In the course of the scenarios Loneliness/Rumination, Party and Café, craving was rated a maximum of 12 times each, and in the Stress scenario a maximum of 14 times. Each scenario included an initial craving rating at the beginning (rating 1). In Loneliness/Rumination this was followed by a craving rating after describing the selected concern (rating 2), a craving rating after ruminating over the concern (rating 3), and a further rating after inspecting the smoking paraphernalia (rating 4). Ratings 5 to 12 consisted of the concluding craving rating phase. In Party, following the initial craving rating, further ratings took place after the first, second, and fourth cigarette offer (rating 2 to 4). Ratings 5 to 12 were again the final craving rating phase. In Café, the initial rating was followed by ratings that took place after each of the three cigarette offers (rating 2 to 4). Ratings 5 to 12 were again the final craving rating phase. In Stress, after the initial rating (rating 1), further ratings were asked for after the first cigarette offer (rating 2), after being informed about the end of the preparation time (rating 3), after the completion of the interview in front of the committee consisting of the presentation and arithmetic task (rating 4), and after the second and third cigarette offer (ratings 5 and 6). Ratings 7 to 14 were the final craving rating phase. In addition to the IPQ, which measured presence after each VR session (i.e. after two VR scenarios), presence ratings were also presented at the end of each VR scenario using the phrase “Between 0 and 100, how strongly do you agree with the following statement: In the computer-generated world, I had the impression of being there. 0 means not at all and 100 means very strongly.” Finally, the question “How confident are you not to smoke in a similar situation?” was used to rate self-efficacy.

*Clinical outcome:* Continuous abstinence was assessed 6 months after the end of treatment adhering to Russel Standard^34^ (RS). RS includes self-reported smoking less than 5 cigarettes during the follow-up period and results of carbon monoxide (CO) measurement below 10 ppm at final follow-up.

### Data reduction and statistical analysis

The lme4 package (v.1.1–34)^35^ was used to analyze linear mixed-effects models in the R environment (v.4.3.1). The random effects structure was determined with model convergence as a requirement and based on Likelihood ratio tests^36^. *F*-tests with Satterthwaite approximations for degrees of freedom^37^ were used to evaluate significance for main effects and interactions in linear mixed-effects models. Post-hoc tests were conducted with the R package emmeans (v.1.8.7)^38^ and corrected for multiple comparisons according to Holm^39^.

For a manipulation check, ratings regarding emotion induction were modeled by including the main effects *emotion* (treatment coding: Worry = reference, Stress, Sociability) and *scenario* (treatment coding: Loneliness/Rumination = reference, Party, Stress, Café) as well as their interaction as fixed effects. Random intercepts were included per participant and s*cenario* was included as random slope per participant in the final model.

Self-efficacy ratings were modeled by including the main effects *scenario* (treatment coding: Loneliness/Rumination = reference), *presentation* (coding: first = reference, second) and *group* (coding: abstainers = reference, relapsed smokers), as well as their interactions as fixed effects. Random intercepts were included per participant in the final model.

Presence ratings were modeled by including the main effects *scenario* (treatment coding: Loneliness/Rumination = reference), *presentation* (coding: first = reference), and *group* (coding: abstainers = reference), as well as their interactions as fixed effects. Random intercepts were included per participant, and s*cenario* and *presentation* were included as random slopes per participant in the final model.

Craving ratings were modeled by including the main effects *scenario* (treatment coding: Loneliness/Rumination = reference), *presentation* (coding: first = reference), *group* (coding: abstainers = reference), and *within-scenario time* (modelled as orthogonal polynomials relating to linear and quadratic effects of test position within a session). The linear effect of *within-scenario time* describes the linear course of craving within a scenario. To have the additional possibility to model the steepness of the increase and decrease of craving within a scenario, we also included the quadratic effect of *within-scenario time* (referred to as *within-scenario time*^*2*^). In addition, the model included the interactions of *presentation*, *scenario*, *group* and *within-scenario time* (linear and quadratic). In the final model, random intercepts were included per participant and random slopes were included for s*cenario*, *presentation* and their interaction. In addition to the main analysis of craving, which included all participants, we conducted an exploratory analysis of craving with the same model, however including only abstinent participants at the time of each VR session. This aimed to account for the fact that some participants were still smoking after the planned quit day (see section Design and Participants above), which might alter craving and bias results.

To analyze the prognostic value of craving during VR-CET on abstinence/relapse, two binomial logistic regression models were calculated in R. In the first model, mean craving across all VR-CET sessions was entered as a predictor. To additionally investigate whether the extent of craving reduction had predictive value, a second model was tested in which the average difference between maximum and last craving rating across VR-CET sessions was entered as a predictor. Likelihood ratio tests were used for overall model estimations. In case of significant model estimations, the model was applied to predict abstinence/relapse based on an optimal cut-off for the craving marker (i.e. simultaneously maximizing specificity and sensitivity), which was calculated with the R package OptimalCutpoints (v.1.1–5) using the Youden index method^40^. In the prediction, participants with values above the optimal threshold were classified as relapsing, participants with values less than or equal to the threshold as abstinent.

The remaining statistical analyses were performed with SPSS 26 (IBM Corp., Armonk, NY, United States). SSQ-b results and presence ratings were non-normally distributed (tested by Kolmogorov–Smirnov tests and visual inspection of the histograms) and non-parametric testing (Wilcoxon test, Spearman correlation) was used. Alpha level was 5% in all statistical analyses.

## Results

### Emotion induction of VR scenarios

For the purpose of a manipulation check we analysed whether induced emotion differed as a function of VR scenario. The linear mixed-effects model revealed a significant interaction between *emotion* and *scenario*, *F*(6, 1491.80) = 106.61, *p* < 0.001, indicating that different scenarios induced different emotions. Follow-up pairwise comparisons indicated that the emotions targeted in each scenario were rated significantly higher than the remaining two emotions (for descriptives see Table 1). In Loneliness/Rumination, worry was significantly higher than stress (b = 8.71, *SE* = 2.35, *t*(1416) = 3.71, *p* < 0.001) and sociability (b = 35.33, *SE* = 2.35, *t*(1417) = 15.01, *p* < 0.001). In Party, sociability was significantly higher than worry (b = 26.47, *SE* = 2.35, *t*(1418) = 11.25, *p* < 0.001) and stress (b = 17.15, *SE* = 2.35, *t*(1417) = 7.30, *p* < 0.001). In the Stress scenario, stress was higher than worry (b = 20.13, *SE* = 2.51, *t*(1416) = 8.03, *p* < 0.001) and sociability (b = 27.69, *SE* = 2.52, *t*(1418) = 10.98, *p* < 0.001). Finally, in Café, sociability was higher than worry (b = 28.87, *SE* = 2.53, *t*(1417) = 11.41, *p* < 0.001) and stress (b = 25.83, *SE* = 2.53, *t*(1417) = 10.21, *p* < 0.001). Supplementary Table S1 provides a full model summary.

**Table 1:** Descriptive results of worry, stress, and sociability ratings for each VR scenario averaged across both presentations. Means (*M*) and standard deviations (*SD*) are given. The rating scale is 0 to 100.

VR scenario	Worry		Stress		Sociability	
	*M*	*SD*	*M*	*SD*	*M*	*SD*
Loneliness/Rumination	41.44	31.83	32.73	29.20	6.14	13.93
Party	19.31	24.04	28.58	27.43	45.57	28.88
Stress	24.64	27.48	44.77	29.59	17.23	20.22
Café	11.02	18.33	14.07	18.91	39.75	28.08

### Self-efficacy in VR scenarios

When asked to rate their confidence about not smoking in a similar (real) situation, participants reported high self-efficacy in the scenarios across both presentations with Café descriptively receiving the highest ratings (*M* = 82.62, *SD* = 60.07), followed by Stress (*M* = 80.00, *SD* = 18.20), Loneliness/Rumination (*M* = 75.88, *SD* = 22.93), and Party (*M* = 70.38, *SD* = 24.16). The linear mixed-effects model yielded neither significant main effects for *scenario, F*(3, 507.23) = 2.05, *p* = 0.101, *presentation, F*(1, 532.10) = 0.98, *p* = 0.323, or *group, F*(1, 98.99) = 2.65, *p* = 0.106, nor significant interactions (*p*s > 0.201). Supplementary Table S2 provides a full model summary.

### Presence during VR

Another analysis investigated the effect of abstinence group, VR scenario, and number of presentation (first, second) on presence ratings in the virtual environment. The linear mixed-effects model revealed significant main effects for *scenario*, *F*(3, 96.11) = 9.24, *p* < 0.001, and *presentation*, *F*(1, 64.37) = 33.28, *p* < 0.001 (for descriptives see Table 2). Main effect *group* and interactions were not significant (*p*s > 0.110). Follow-up tests for the main effect *scenario* indicated that Loneliness/Rumination received significantly higher presence ratings than Stress (b = 10.37, *SE* = 2.84, *t*(84.8) = 3.66, *p* = 0.002) and Café (b = 8.73, *SE* = 2.55, *t*(82.1) = 3.43, *p* = 0.003), whereas Loneliness/Rumination and Party did not differ significantly (b = − 1.76, *SE* = 2.29, *t*(84.4) = − 0.77, *p* = 0.853). Party received significantly higher presence ratings than Stress (b = 12.13, *SE* = 2.46, *t*(81.9) = 4.93, *p* < 0.001) and Café (b = 10.49, *SE* = 2.50, *t*(82.4) = 4.20, *p* < 0.001). Stress and Café did not differ significantly (b = − 1.64, *SE* = 2.05, *t*(76.5) = − 0.77, *p* = 0.853). With respect to the main effect *presentation*, follow-up tests indicated that presence decreased significantly from the first to the second run of each scenario (Loneliness/Rumination: b = 10.55, *SE* = 2.92, *t*(233) = 3.62, *p* < 0.001; Party: b = 11.92, *SE* = 2.90, *t*(235) = 4.11, *p* < 0.001; Stress: b = 11.87, *SE* = 3.20, *t*(251) = 3.71, *p* < 0.001; Café: b = 9.05, *SE* = 3.21, *t*(253) = 2.82, *p* = 0.005). Supplementary Table S3 provides a full model summary.

**Table 2:** Descriptive results of single item presence ratings for each VR scenario and presentation. Means (*M*) and standard deviations (*SD*) are given. The rating scale is 0 to 100.

VR scenario	1st presentation		2nd presentation	
	*M*	*SD*	*M*	*SD*
Loneliness/Rumination	57.36	27.94	47.38	28.09
Party	59.98	25.82	49.66	27.84
Stress	49.62	27.15	42.56	28.52
Café	49.36	28.80	46.55	26.67

To assess presence across VR sessions, we furthermore used the IPQ (range from − 3 to 3). Presence as measured with the IPQ was M = 0.18 (SD = 1.06) in session 1 of VR-CET, M = − 0.31 (SD = 1.17) in session 2, M = − 0.36 (SD = 1.29) in session 3, and M = − 0.31 (SD = 1.28) in session 4. To validate presence ratings for each scenario, we averaged the single-item ratings of each scenario presented within one VR session and compared these values to the IPQ scores of each VR session by conducting Spearman correlation coefficients. This yielded significant and large positive correlations between the presence measured by single-item ratings and the presence measured via IPQ, indicating the validity of the single-item ratings (session 1: *r*(95) = 0.70, *p* < 0.001; session 2: *r*(78) = 0.79, *p* < 0.001; session 3: *r*(62) = 0.83, *p* < 0.001; session 4: *r*(60) = 0.82, *p* < 0.001).

### Tolerability of VR scenarios

Across the scenarios, symptoms of simulator sickness such as headache (M = 0.33, SD = 0.46), nausea (M = 0.18, SD = 0.40), and vertigo (M = 0.56, SD = 0.61) were reported to a low degree, indicating good tolerability of the VR scenarios. The scale for each symptom category ranged from 0 (no symptoms at all) to 3 (severe symptoms). Future abstainers and relapsed smokers did not differ significantly with regard to the severity of symptoms (*p*s > 0.455).

### Course of craving during VR-CET

The linear mixed-effects model for craving ratings yielded significant main effects of *group*, *presentation*, *scenario*, *within-scenario time*, and *within-scenario time*^*2*^, significant two-way interactions of *presentation* × *scenario*, *presentation* × *within-scenario time*, *presentation* × *within-scenario time*^*2*^, and *scenario* × *within-scenario time*, and significant three-way interactions of *presentation* × *scenario* × *within-scenario time*, *presentation* × *group* × *within-scenario time*, and *scenario* × *group* × *within-scenario time*^*2*^ (statistical results shown in Table 3). All other effects were not significant.

**Table 3:** Significant effects of *F*-tests with Satterthwaite approximations for degrees of freedom for the linear mixed-effects model for craving ratings.

Effect	*df*_*Num*_	*df*_*Den*_	*F*	*p*
Group	1	93.5	10.93	.001
Presentation	1	80.9	46.78	< .001
Scenario	3	79.7	10.28	< .001
Within-scenario time	1	6589.5	277.46	< .001
Within-scenario time^2^	1	6577.3	48.00	< .001
Presentation × scenario	3	70.9	4.94	.004
Presentation × within-scenario time	1	6596.4	25.69	< .001
Presentation × within-scenario time^2^	1	6580.2	8.01	.005
Scenario × within-scenario time	3	6607.5	12.21	< .001
Presentation × scenario × within-scenario time	3	6608.4	11.55	< .001
Presentation × group × within-scenario time	1	6596.4	5.22	.022
Scenario × group × within-scenario time^2^	3	6582.1	3.14	.024

**Figure 3:**
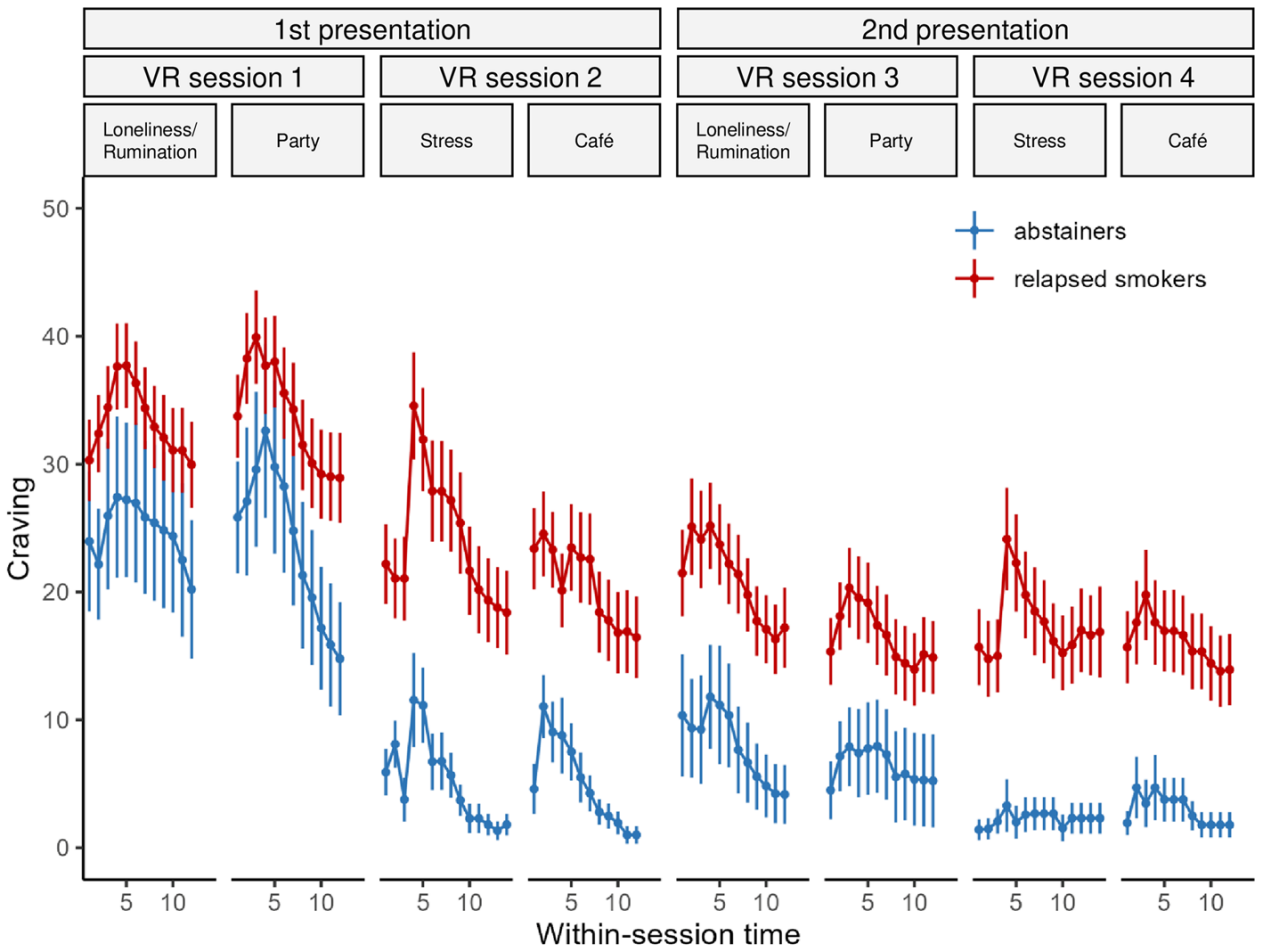
Within and between scenario craving ratings during the course of the VR-CET for abstainers and relapsed smokers. Fifth and tenth craving rating is marked on the x-axis for each scenario. Error bars show standard errors.

The main effect *group* indicated that smokers who relapse experience stronger craving throughout VR-CET than those who maintain abstinence, thus confirming our hypothesis (see Fig. 3 for an overview of craving ratings during the course of the VR-CET; for a presentation of craving by scenario, including specific events within the scenarios, see supplementary Fig. S1).

In the following, we first describe the additional effects that include the factor *group*, second the effects that include the factor *presentation* (without *group*), and third the effects that include the factor *scenario* (without *group* and *presentation*).

Post hoc tests for the three-way interaction of *presentation* × *group* × *within-scenario time* indicated that, relative to relapsed smokers, abstainers tended to have stronger linear decreases in craving in the first presentation of scenarios, b = − 3.43, *SE* = 1.62, *t*(6580) = − 2.11, *p* = 0.069 (*p* = 0.035 without adjustment for multiple comparisons), whereas this effect was not significant for the second presentation (b = 2.32, *SE* = 1.92,* t*(6601) = 1.21, *p* = 0.228). Post hoc tests for the three-way interaction of *scenario* × *group* × *within-scenario time*^*2*^ indicated that the quadratic trend of *within-scenario time* was stronger in relapsed smokers relative to abstainers in the stress scenario, b = 4.95, *SE* = 1.86, *t*(6571) = 2.67, *p* = 0.031, whereas this effect was not significant in the remaining three scenarios (*p*s > 0.320). This means that relapsed smokers showed more craving directly following the stress induction.

Post hoc tests with respect to the interaction of *presentation* × *scenario* indicated that craving during the first presentation was significantly higher compared to the second presentation for all scenarios (Loneliness/Rumination: b = 16.35, *SE* = 2.75, *t*(86.8) = 5.95, *p* < 0.001; Party: b = 17.92, *SE* = 2.45, *t*(83.7) = 7.32, *p* < 0.001; Stress: b = 6.18, *SE* = 2.37, *t*(65.2) = 2.60, *p* = 0.023; Café: b = 5.02, *SE* = 2.24, *t*(70.4) = 2.24, *p* = 0.028) and that this decrease was more pronounced in the scenarios Loneliness/Rumination and Party than in Stress and Café (Loneliness/Rumination compared with Stress: b = 10.17, *SE* = 3.61, *t*(72.6) = 2.82, *p* = 0.019; Loneliness/Rumination compared with Café: b = 11.34, *SE* = 3.48, *t*(76.9) = 3.26, *p* = 0.007; Party compared with Stress: b = 11.74, *SE* = 3.29, *t*(69.4) = 3.57, *p* = 0.003; Party compared with Café: b = 12.91, *SE* = 3.28, *t*(70.8) = 3.93, *p* = 0.001; no significant differences for comparisons of Loneliness/Rumination and Party, *p* = 1.000, and Stress and Café, *p* = 1.000). The interactions of *presentation* × *within-scenario time* and *presentation* × *within-scenario time*^*2*^, indicate that the linear and quadratic course of craving differed across presentations. Post hoc comparisons revealed that the second presentation of scenarios resulted in smaller linear decreases in craving, b = − 6.37, *SE* = 1.26, *t*(6596) = − 5.07, *p* < 0.001, and in terms of the quadratic effect, in a flatter increase and decrease of craving, b = 3.46, *SE* = 1.22, *t*(6580) = 2.83, *p* = 0.005. In addition, the three-way interaction of *presentation* × *scenario* × *within-scenario time* indicates that the *presentation* × *within-scenario time* effect described above, differed between scenarios. Whereas in Loneliness/Rumination, the difference in the linear course of craving between first and second presentation was not significant, b = 4.84, *SE* = 2.58, *t*(6583) = 1.88, *p* = 0.060, for all three remaining scenarios, stronger linear decreases of craving were observed in the first compared to the second presentation (Party: b = − 16.67, *SE* = 2.62, *t*(6597) = − 6.36, *p* < 0.001; Stress: b = − 7.24, *SE* = 1.88, *t*(6620) = − 3.84, *p* < 0.001; Café: b = − 6.43, *SE* = 2.86, *t*(6608) = − 2.25, *p* = 0.060).

The interaction of *scenario* × *within-scenario time* results from a more negative linear decrease of craving in Party compared to Stress, b = − 9.33, *SE* = 1.62, *t*(6611) = − 5.78, *p* < 0.001, in Party compared to Café, b = − 5.20, *SE* = 1.94, *t*(6609) = − 2.68, *p* = 0.030, in Loneliness/Rumination compared to Stress, b = − 5.87, *SE* = 1.60, *t*(6607) = − 3.68, *p* = 0.001, and in Café compared to Stress, b = − 4.13, *SE* = 1.71, *t*(6621) = − 2.42, *p* = 0.0495. Finally, the main effect *scenario* results from higher craving in Loneliness/Rumination than in Stress, b = 7.57, *SE* = 1.90, *t*(79.7) = 3.98, *p* < 0.001, in Loneliness/Rumination than in Café, b = 9.91, *SE* = 1.86, *t*(73.5) = 5.33, *p* < 0.001, in Party than in Stress, b = 5.63, *SE* = 1.77, *t*(80.8) = 3.18, *p* = 0.006, and in Party than in Café, b = 7.97, *SE* = 1.55, *t*(75.6) = 5.13, *p* < 0.001. Loneliness/Rumination and Party (*p* = 0.204), and Stress and Café (*p* = 0.129) did not differ significantly. Supplementary Table S4 provides a full model summary.

An identical analysis, including only abstinent participants at the time of each VR session, is detailed in the supplementary materials (see Table S5 and Fig. S2) and yielded basically similar results.

### Predictive ability of craving on abstinence

Two binomial logistic regressions with mean craving across all VR-CET sessions and with the difference between maximum craving and the final craving rating averaged across all VR-CET sessions as predictors for the likelihood of abstinence at 6-month follow-up were performed. The overall model evaluation for the first model using mean craving as a predictor was statistically significant, χ^2^(1) = 12.69, *p* < 0.001, R^2^_N_ = 0.176. Mean craving was positively associated with relapse (*β* = 0.058, *SE* = 0.019, *z* = 2.98, *p* = 0.003, OR = 1.06). Applying Youden’s criterion yielded an optimal cut-off for mean craving of *M* > 12.63 for predicting relapse. This way, the model correctly classified 17 of 24 future abstainers (70.8% specificity) and 55 of 78 future relapsing smokers (70.5% sensitivity) resulting in overall 72 of 102 correct classifications (70.6% accuracy).

The overall model evaluation for the second model using the difference between maximum and final craving rating as a predictor was statistically not significant, χ^2^(1) = 0.691, *p* = 0.406, R^2^_N_ = 0.010.

## Discussion

To the best of our knowledge, the present study is the first to analyze craving ratings during VR-CET prospectively with regard to subsequent abstinent and relapsing smokers. In addition, we investigated further parameters of the VR-CET scenarios, namely emotion induction, self-efficacy, presence, and tolerability, which might influence their efficacy.

The analysis of craving across the virtual exposure sessions with respect to future abstainers and relapsed smokers revealed three interesting patterns: First, future relapsed smokers showed generally higher craving than future abstainers which also predicted relapse. Second, relapsed smokers showed stronger increases in craving elicited by the Stress scenario than abstainers. Third, abstainers tended to down-regulate craving faster within the first presentation of scenarios. Regarding the first aspect, smokers that relapsed within 6 months after end of treatment showed higher craving levels than future abstainers during VR-CET. While craving generally decreased across VR-CET (which is in line with previous reports^21,22^ and also consistent with the CE rational), it remained consistently higher in relapsing smokers. This is in line with previous findings from the neuroimaging literature, which found increased brain activation in substance use disorders with respect to substance-related cues in cue-reactivity tasks in relapsing individuals relative to individuals who remained abstinent^41^. Additionally, our results are consistent with an analysis of several nicotine replacement therapy (NRT) trials, which found that low craving was associated with a higher likelihood of sustained abstinence^42^. The differences found in craving between future abstainers and relapsing smokers appear even more relevant as the two groups did not significantly differ in any other smoking parameter such as cigarettes per day, Fagerström score or carbon monoxide levels. Furthermore, high craving was a significant prognostic factor for higher relapse probability. This effect was small, however, demonstrating the need for further research in identifying additional predictors of relapse. As reviewed by Wray et al.^6^, cue-induced craving during classical cue-reactivity assessment was only rarely a significant predictor of treatment outcome in previous studies, which contrasts our finding. One reasons for this difference could be, in accordance with the argumentation of Tiffany et al.^2^, the higher correspondence of the situation of the craving assessment and real-life situations by using VR. Therefore, the predictive effect of craving may be still larger if presence in VR could be further increased. Another reason could be that the VR-CET sessions took place after the smoking cessation program’s planned quit date, thus craving during VR-CET was assessed in a phase in which the majority of the participants had achieved abstinence. This could have been beneficial for the prognostic capacity of craving, as it was argued that the combination of substance-related cues and abstinence is better suited for detecting craving as an indicator of relapse than during periods of nicotine saturation^43^. Taken together, the first pattern implies that participants with high craving should be considered a high-risk group for relapse and therapy should be individualized accordingly. This could include both increased discussion of coping strategies for craving in the CBT component of treatment and possibly a stronger recommendation of additional NRT.

Second, future relapsed smokers showed a stronger craving response to the stress induction in the Stress scenario than future abstainers. This suggests that reducing the craving response to stress should be an important therapeutic goal in smoking cessation, which is in line with previous research underscoring the particular role of stress by reporting that stress increases cigarette craving and promotes relapse^44,45^. The fact that craving amplitudes generally attenuate across presentations is consistent with the rational of CE and suggests that reducing the craving response to stress may be achieved by further runs of the Stress scenario. In addition, participants with a strong craving response to stress might benefit from an intensification of stress management and the development of further coping strategies as part of the CBT component of smoking cessation. Future trials should examine whether these considerations translate into higher efficacy of VR-CET.

Third, the linear decrease in craving within a scenario significantly differed as a function of whether a scenario was presented for the first or the second time and whether participants would remain abstinent. Thus, the linear within-scenario decrease in craving may affect treatment success as future abstainers tended to down-regulate their craving more quickly. However, this finding should be treated with caution for three reasons. First, whereas there was a trend of faster craving regulation by future abstainers in the first presentation of scenarios, this was not the case with respect to the second presentation (although floor effects in the second presentation may have contributed). Second, this three-way interaction did not show up significantly in the supplementary analysis that included only abstainers at the time of VR-CET as linear within-scenario craving reductions of future abstainers and relapsed smokers were more similar than in the main analysis. And third, the importance of the role of within-scenario craving decrease is reduced by the fact that the regression analysis examining the difference from maximum craving to the last craving rating did not significantly predict abstinence.

As part of the VR-CET, participants were confronted with a variety of smoking related situations. The Loneliness/Rumination scenario featured an intensive cue exposure with detailed observation of smoking paraphernalia while simultaneously inducing worry. The Party scenario, like the Stress and Café scenario, included interactions with smoking characters who offered cigarettes to the participants, whereby the presence of alcoholic beverages and witnessing a relapse were distinctive features. The stress scenario required participants to complete a job application presentation and an unannounced math task while smoking was presented as a beneficial coping strategy and reward by other characters. Finally, the Café scenario included coffee as a typical smoking associated stimulus and a conversation of smokers about smoking as a feeling of freedom and enjoyment in life. These scenarios were able to induce the specific intended emotions (worry, stress, sociability), which demonstrates the usefulness of VR for CET as it enables the presentation of substance-related cues in emotional contexts thus increasing ecological validity compared to traditional CET paradigms. This is in line with previous research that found VR to be generally effective in inducing emotions^46^ and showed that different emotional states can be induced with relative distinctness^47^. With regard to the general decline in craving across the VR scenarios, we can only speculate whether certain elements of the scenarios were particularly important or whether it was the variety that was decisive (see also limitations below on order effects). However, the Stress scenario stands out because of the most pronounced increase in craving after the stress task, which only disappeared for future abstainers in the second run. Particularly the elements of stress relief and reward after completing a task, which are not present in the other scenarios, may lead to this sharper increase in craving. This notion is in line with Fagerström^48^ who emphasizes the importance of the rewarding function of smoking.

In terms of self-efficacy, participants reported high levels of confidence in not smoking in similar situations in the future for all scenarios without significant differences between future abstainers and relapsed smokers. This conviction is understandable because treatment-seeking participants must exhibit a certain level of self-efficacy expectancy to enroll in treatment in the first place, because the majority of participants had already achieved abstinence at the time of VR-CET, and because the question about self-efficacy was asked at the end of the scenario, which virtually had just been successfully completed without smoking. Nevertheless, the results suggest that therapists should remain cautious and not base therapeutic actions on self-efficacy as the majority of participants later relapsed, although self-efficacy was high during VR-CET.

With respect to presence (the sense of being there), VR scenarios generated moderate levels of presence. Party and Loneliness/Rumination induced significantly higher levels of presence than Café and Stress, and presence generally decreased in the second compared to the first presentation of scenarios. Both measures of presence (single item rating and IPQ) were highly correlated, indicating validity of the single item measure. Presence is regarded as an indicator of felt similarity to real-life situations^9^, therefore high presence is desirable to generalize the learning experiences within VR to real life. Higher presence could be achieved through the ongoing graphical progress in the field of VR^49^. In addition, tactile (e.g. feeling a real table of a café in front of oneself), olfactory (e.g. smell of tobacco, smoke, coffee, barbecue), and auditory (audio renderings which simulate room acoustics and spatial sound) sensory modalities could be added to increase the observed moderate presence in future applications. The differences between the scenarios in terms of experienced presence probably mainly reflect the decrease in presence over several sessions, since the earlier scenarios generated higher presence than the later scenarios. It is a known phenomenon that stronger emotional experience is associated with higher presence, which is explained by increased arousal^49^. In this respect, the reduction in presence over time may be explained by decreasing arousal with repeated VR-CET through habituation processes. Although presented first, the relatively high level of presence in Loneliness/Rumination is somewhat surprising given that it is the only scenario without interaction with virtual agents. However, in this scenario, the aforementioned auditory aspect might have additionally contributed to the experienced presence through the sound of rain and music. It is noticeable that despite high emotional arousal in the Stress scenario, the experience of presence was comparatively lower. We suppose that there may have been a lower felt similarity to real-life situations as participants are likely to be exposed to other stressful situations in their daily lives. This intensive stress induction, which tends to be more distant from everyday life, nonetheless led to craving reductions over the course of the treatment and enabled future abstainers and relapsing smokers to be distinguished. Future studies could investigate whether the approach of the present study or the induction of a more moderate but everyday stress would result in higher presence and be more favorable for use in VR-CET. While presence is an important parameter in VR research, it will often not be feasible to assess presence with questionnaires (e.g. in case of multiple ratings) and these have the potential disadvantage of assessing presence retrospectively when immersion in VR has already ended. In this regard, our results support the further use of single item ratings during VR as a more economical alternative.

Finally, regarding tolerability, VR-CET sessions were well tolerated with no evidence that this could account for differences in treatment success.

An important limitation of the current study is that the data analyzed derive from a treatment study primarily designed to evaluate the efficacy of VR-CET as an additional intervention to group CBT compared with a relaxation procedure and group CBT. In this respect, we cannot rule out the possibility that the observed craving reductions are due to causes other than VR-CET, such as effects of CBT alone, or to the simple progression of time. These explanations nevertheless seem unlikely as they cannot explain the within scenario craving reductions, because VR-CET alone led to craving reductions in a previous study^21^, and because cue-induced craving usually increases and not decreases following abstinence, a phenomenon called incubation of craving^50^. An additional limitation is the likely occurrence of order effects with respect to presence and craving as scenarios were presented in a uniform order without counterbalancing. As an example, despite the significantly higher craving in Party than in Café, we cannot conclude that this is due to content differences of the scenarios (the presence of alcoholic beverages produces higher craving than coffee could be such a content interpretation), because Café was chronologically always presented after Party. We therefore refrained from interpreting such direct scenarios comparisons. Future studies could use our scenarios in a counterbalanced design to further examine these content differences.

In conclusion, we found evidence that smokers who relapse within 6 months exhibit higher levels of craving during VR-CET than do smokers who maintain abstinence and that craving during VR-CET predicts relapse. Furthermore, we found that craving responses evoked by the Stress scenario were discriminative with respect to future abstinence as relapsing smokers reacted with stronger craving. Attempts to improve VR-CET and ultimately smoking cessation outcomes, should aim to identify smokers who are more at risk of relapse based on their persistently high craving levels and individualize smoking cessation accordingly. Additionally, particular emphasis should be placed on the strength of the craving response to stress, which may be lowered by further therapeutic efforts applying stress-inducing scenarios.

### Supplementary Information

Supplementary Information.

## Data Availability

The datasets analyzed during the current study are available from the corresponding author on reasonable request.
