# Supplementary material for: Cigarette craving in virtual reality cue exposure in abstainers and relapsed smokers
Source: Sci Rep. 2024 Mar 30;14:7538. doi: 10.1038/s41598-024-58168-7 (PMC10980682; doi:10.1038/s41598-024-58168-7)
Supplement: Supplementary file 1 — Supplementary Information. [file 41598_2024_58168_MOESM1_ESM.pdf]

Supplementary Material:

Cigarette craving in virtual reality cue exposure in abstainers and relapsed smokers

Benedikt Schröder[1*], Agnes Kroczek[2,3], Leon O. H. Kroczek[1], Ann-Christine Ehlis[2,4], Anil Batra[2,3,4] and Andreas Mühlberger[1]

[1]Department for Psychology, Clinical Psychology and Psychotherapy, University of Regensburg, Germany

[2] Department of Psychiatry and Psychotherapy, Tübingen Center for Mental Health (TüCMH), University Hospital Tübingen, Germany

[3] Department of Psychiatry and Psychotherapy, Tübingen Center for Mental Health (TüCMH), Section for Addiction Research and Medicine University Hospital Tübingen, Germany

[4] German Center for Mental Health (DZPG)

Model summaries of linear mixed-effects models

Emotion Induction

Table S1. Model summary for linear mixed-effects model for emotion induction with formula: Emotion rating ~ scenario * emotion + (1 + scenario | subject).

Predictors	*Estimates*	*SE*	*df*	*t*	*p*
Intercept	42.50	2.36	197.24	17.99	< .001
Scenario Party	-22.41	2.72	349.72	-8.25	< .001
Scenario Stress	-16.78	2.96	279.72	-5.68	< .001
Scenario Café	-30.75	2.79	370.71	-11.01	< .001
Emotion Stress	-8.71	2.35	1490.88	-3.71	< .001
Emotion Sociability	-35.34	2.35	1492.19	-15.01	< .001
Scenario Party × Emotion Stress	18.03	3.32	1491.22	5.43	< .001
Scenario Stress × Emotion Stress	28.84	3.43	1490.88	8.40	< .001
Scenario Café × Emotion Stress	11.75	3.45	1490.88	3.41	< .001
Scenario Party × Emotion Sociability	61.81	3.33	1492.64	18.57	< .001
Scenario Stress × Emotion Sociability	27.77	3.45	1492.58	8.05	< .001

Self-efficacy

Table S2. Model summary for linear mixed-effects model for self-efficacy with formula: Self-efficacy rating ~ scenario * presentation * group + (1 | subject).

**Predictors**	***Estimates***	***SE***	***df***	***t***	***p***
Intercept	79.79	7.07	530.94	11.29	< .001
Scenario Party	-7.50	9.25	498.26	-0.81	.418
Scenario Stress	4.31	9.48	502.18	0.46	.649
Scenario Café	-0.69	9.48	502.18	-0.07	.942
Presentation second	9.98	9.74	506.23	1.03	.306
Group relapsed	-8.46	8.17	533.24	-1.04	.301
Scenario Party × Presentation second	0.25	13.72	498.26	0.02	.985
Scenario Stress × Presentation second	-7.73	14.30	514.36	-0.54	.589
Scenario Café × Presentation second	-5.09	14.30	514.36	-0.36	.722
Scenario Party × Group relapsed	2.27	10.70	499.02	0.21	.832
Scenario Stress × Group relapsed	1.78	11.08	507.01	0.16	.873
Scenario Café × Group relapsed	17.15	11.10	507.84	1.55	.123
Presentation second × Group relapsed	-7.24	11.53	511.12	-0.63	.530
Scenario Party × Presentation second × Group relapsed	0.98	16.21	498.59	0.06	.952
Scenario Stress × Presentation second × Group relapsed	6.15	16.88	516.43	0.36	.716
Scenario Café × Presentation second × Group relapsed	-9.03	16.90	517.29	-0.53	.593

Presence

Table S3 Model summary for linear mixed-effects model for presence ratings with formula: Presence rating ~ scenario * presentation * group + (1 + scenario + presentation | subject).

**Predictors**	***Estimates***	***SE***	***df***	***t***	***p***
Intercept	54.88	5.59	105.65	9.82	< .001
Scenario Party	3.17	4.75	171.64	0.67	.506
Scenario Stress	-14.56	5.50	129.61	-2.65	.009
Scenario Café	-11.29	5.05	144.64	-2.24	.027
Presentation second	-10.15	4.86	253.24	-2.09	.038
Group relapsed	3.05	6.44	107.24	0.47	.637
Scenario Party × Presentation second	-5.62	6.15	226.64	-0.91	.362
Scenario Stress × Presentation second	-3.51	6.63	247.90	-0.53	.597
Scenario Café × Presentation second	-4.78	6.63	259.57	-0.72	.472
Scenario Party × Group relapsed	-1.45	5.50	173.58	-0.26	.793
Scenario Stress × Group relapsed	9.70	6.44	134.91	1.51	.134
Scenario Café × Group relapsed	3.62	5.94	151.28	0.61	.543
Presentation second × Group relapsed	-0.82	5.80	258.24	-0.14	.888
Scenario Party × Presentation second × Group relapsed	8.49	7.32	236.45	1.16	.247
Scenario Stress × Presentation second × Group relapsed	4.38	7.89	258.25	0.56	.580
Scenario Café × Presentation second × Group relapsed	12.56	7.91	268.41	1.59	.114

Craving

Table S4. Model summary for linear mixed-effects model for craving ratings with formula: Craving rating ~ presentation * scenario * group * (within-scenario time + within-scenario time^2) + (1 + scenario * presentation | subject).

**Predictors**	***Estimates***	***SE***	***df***	***t***	***p***
Intercept	23.61	5.33	93.54	4.43	< .001
Presentation second	-18.17	4.71	85.44	-3.86	< .001
Scenario Party	-3.16	4.02	92.23	-0.79	.434
Scenario Stress	-17.06	5.02	74.93	-3.40	.001
Scenario Café	-18.14	4.77	78.41	-3.80	< .001
Group relapsed	9.45	6.14	94.50	1.54	.127
within-scenario time	-8.64	2.87	6565.62	-3.01	.003
within-scenario time^2	8.97	2.81	6565.62	3.19	.001
Presentation second × Scenario Party	2.77	4.51	81.81	0.61	.541
Presentation second × Scenario Stress	13.92	6.16	70.79	2.26	.027
Presentation second × Scenario Café	14.48	5.95	75.95	2.43	.017
Presentation second × Group relapsed	3.62	5.52	88.60	0.66	.514
Scenario Party × Group relapsed	2.65	4.65	92.90	0.57	.570
Scenario Stress × Group relapsed	9.02	5.86	77.38	1.54	.127
Scenario Café × Group relapsed	5.38	5.57	80.85	0.97	.337
Presentation second × within-scenario time	-4.81	4.39	6586.72	-1.10	.273
Presentation second × within-scenario time^2	-6.14	4.27	6574.28	-1.44	.151
Scenario Party × within-scenario time	-20.14	4.10	6568.51	-4.91	< .001
Scenario Stress × within-scenario time	-0.68	3.51	6575.03	-0.19	.846
Scenario Café × within-scenario time	-6.13	4.31	6591.48	-1.42	.155
Scenario Party × within-scenario time^2	1.56	4.01	6570.89	0.39	.697
Scenario Stress × within-scenario time^2	-5.66	3.46	6565.94	-1.64	.102
Scenario Café × within-scenario time^2	-6.38	4.17	6573.94	-1.53	.126
Group relapsed × within-scenario time	-0.90	3.32	6565.68	-0.27	.786

Group relapsed × within-scenario time^2	0.39	3.25	6565.76	0.12	.905
Presentation second × Scenario Party × Group relapsed	-6.49	5.27	85.19	-1.23	.221
Presentation second × Scenario Stress × Group relapsed	-6.39	7.24	73.39	-0.88	.380
Presentation second × Scenario Café × Group relapsed	-5.64	7.00	79.17	-0.81	.423
Presentation second × Scenario Party × within-scenario time	28.33	6.27	6601.78	4.52	< .001
Presentation second × Scenario Stress × within-scenario time	13.57	5.46	6609.45	2.49	.013
Presentation second × Scenario Café × within-scenario time	14.32	6.61	6606.45	2.17	.030
Presentation second × Scenario Party × within-scenario time^2	-0.99	6.09	6583.89	-0.16	.870
Presentation second × Scenario Stress × within-scenario time^2	3.54	5.33	6574.15	0.66	.507
Presentation second × Scenario Café × within-scenario time^2	5.32	6.39	6585.33	0.83	.406
Presentation second × Group relapsed × within-scenario time	-0.07	5.15	6582.71	-0.01	.989
Presentation second × Group relapsed × within-scenario time^2	0.07	5.03	6573.15	0.01	.988
Scenario Party × Group relapsed × within-scenario time	11.83	4.73	6568.44	2.50	.013
Scenario Stress × Group relapsed × within-scenario time	1.02	4.08	6573.33	0.25	.802
Scenario Café × Group relapsed × within-scenario time	4.46	5.00	6587.18	0.89	.372
Scenario Party × Group relapsed × within-scenario time^2	-7.06	4.64	6570.40	-1.52	.128
Scenario Stress × Group relapsed × within-scenario time^2	5.85	4.03	6566.39	1.45	.146
Scenario Café × Group relapsed × within-scenario time^2	-3.43	4.86	6573.40	-0.71	.480
Presentation second × Scenario Party × Group relapsed × within-scenario time	-13.63	7.35	6596.80	-1.86	.064
Presentation second × Scenario Stress × Group relapsed × within-scenario time	-2.97	6.38	6603.43	-0.47	.641
Presentation second × Scenario Café × Group relapsed × within-scenario time	-6.09	7.70	6601.14	-0.79	.429
Presentation second × Scenario Party × Group relapsed × within-scenario time^2	5.04	7.16	6581.41	0.70	.482
Presentation second × Scenario Stress × Group relapsed × within-scenario time^2	-2.65	6.25	6573.09	-0.42	.672

Craving course by scenario

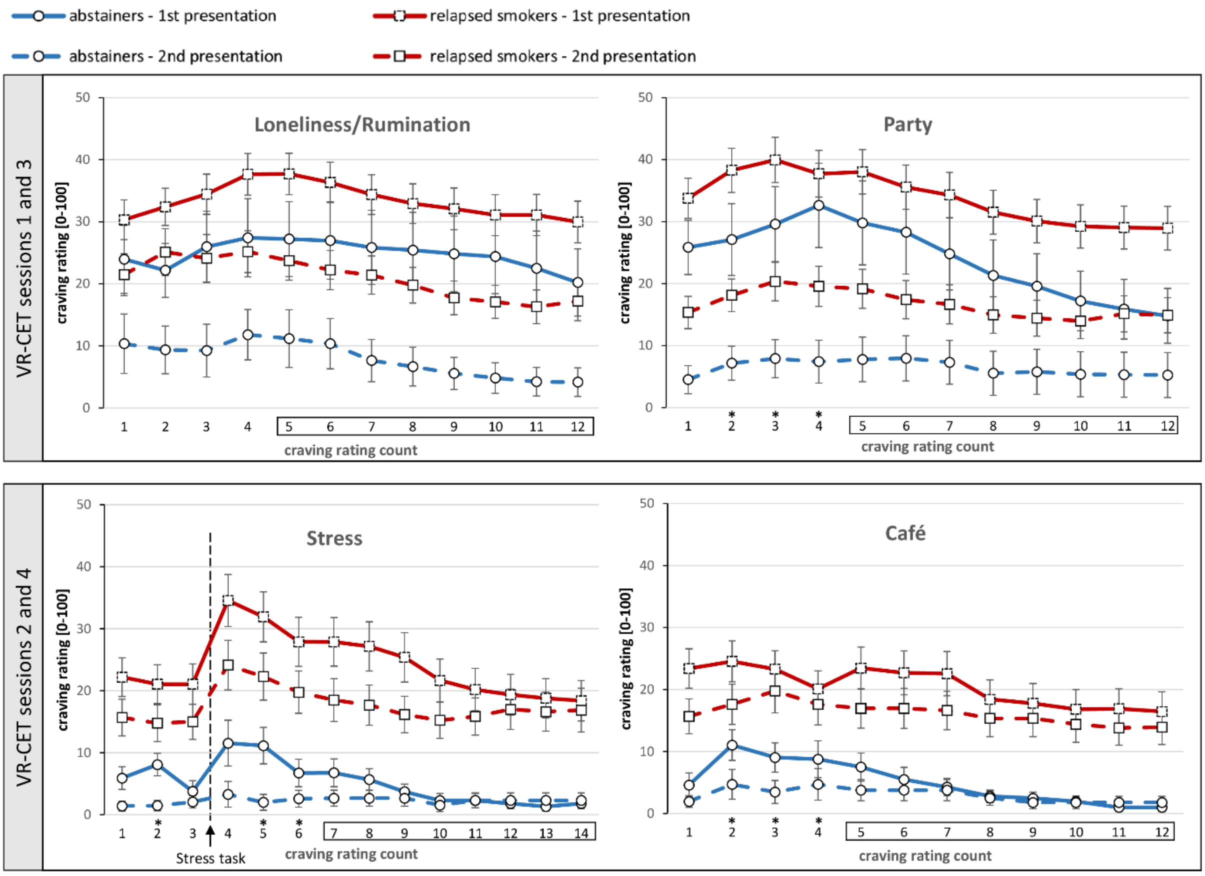

Figure S1. Craving ratings during the course of the VR scenarios for abstainers and relapsed smokers. Error bars show standard errors. Asterisks indicate ratings after cigarette offers. Frames around the last 8 numbers of the x-axis indicate the final craving rating phase.

Course of craving for only abstinent participants at the time of VR-CET sessions

Table S5. Significant effects of *F*-tests with Satterthwaite approximations for degrees of freedom for the linear mixed-effects model for craving ratings of abstinent participants at the time of VR-CET sessions.

Effect	df_{Num}	df_{Den}	F	p
group	1	84.1	6.84	.011
presentation	1	72.7	40.39	< .001
scenario	3	69	9.53	< .001
within-scenario time	1	5681.7	310.71	< .001
within-scenario time^2	1	5672.7	55.11	< .001
presentation × scenario	3	63.3	5.73	.002
presentation × within-scenario time	1	5687.6	40.64	< .001
presentation × within-scenario time^2	1	5675.2	9.79	.002
scenario × within-scenario time	3	5696.7	15.64	< .001
presentation × scenario × within-scenario time	3	5697.4	11.75	< .001
scenario × group × within-scenario time^2	3	5677.5	4.45	.004

The main effect *group* indicated that smokers who relapse experience stronger craving throughout VR-CET than those who maintain abstinence, thus confirming our hypothesis (see Figure S1 for an overview of craving ratings during the course of the VR-CET). In the following, we first describe the additional effect that includes the factor *group*, second the effects that include the factor *presentation* (without *group*), and third the effects that include the factor *scenario* (without *group* and *presentation*).

Post hoc tests for the three-way interaction of *scenario* × *group* × *within-scenario time*^{2} indicated that the quadratic trend of *within-scenario time* was stronger in relapsed smokers relative to abstainers in the stress scenario, b = 5.96, *SE* = 1.88, *t*(5669) = 3.18, *p* = .006, whereas this effect was not significant in the remaining three scenarios (*p*s > .117). This means that relapsed smokers showed more craving directly following the stress induction.

Post hoc tests with respect to the interaction of *presentation* × *scenario* indicated that craving during the first presentation was significantly higher compared to the second presentation for Loneliness/Rumination, Party and Stress (Loneliness/Rumination: b = 17.57, *SE* = 3.09, *t*(74.9) = 5.69, *p* < .001; Party: b = 18.82, *SE* = 2.76, *t*(72.1) = 6.82, *p* < .001; Stress: b = 6.02, *SE* = 2.45, *t*(48.7) = 2.46, *p* = .035), whereas there was only a trend in the same direction for Café, b = 4.22, *SE* = 2.32, *t*(50.2) = 1.82, *p* = .075. In addition, the decrease of craving was more

pronounced in the scenarios Loneliness/Rumination and Party than in Stress and Café (Loneliness/Rumination compared with Stress: b = 11.54, *SE* = 3.94, *t*(64.0) = 2.93, *p* = .014; Loneliness/Rumination compared with Café: b = 13.35, *SE* = 3.69, *t*(64.5) = 3.62, *p* = .003; Party compared with Stress: b = 12.79, *SE* = 3.67, *t*(56.3) = 3.49, *p* = .004; Party compared with Café: b = 14.60, *SE* = 3.51, *t*(56.1) = 4.16, *p* < .001; no significant differences for comparisons of Loneliness/Rumination and Party, *p* = .666, and Stress and Café, *p* = .554). The interactions of *presentation* × *within-scenario time* and *presentation* × *within-scenario* $time^2$, indicate that the linear and quadratic course of craving differed across presentations. Post hoc comparisons revealed that the second presentation of scenarios resulted in smaller linear decreases in craving, b = -8.19, *SE* = 1.28, *t*(5688) = -6.38, *p* < .001, and in terms of the quadratic effect, in a flatter increase and decrease of craving, b = 3.92, *SE* = 1.25, *t*(5675) = 3.13, *p* = .002. In addition, the three-way interaction of *presentation* × *scenario* × *within-scenario time* indicates that the *presentation* × *within-scenario time* effect described above, differed between scenarios. Whereas in Loneliness/Rumination, the difference in the linear course of craving between first and second presentation was not significant, b = 3.48, *SE* = 2.66, *t*(5676) = 1.30, *p* = .192, for all three remaining scenarios, stronger linear decreases of craving were observed in the first presentation (Party: b = -18.96, *SE* = 2.72, *t*(5691) = -6.98, *p* < .001; Stress: b = -9.27, *SE* = 1.89, *t*(5707) = -4.90, *p* < .001; Café: b = -8.00, *SE* = 2.88, *t*(5699) = -2.78, *p* = .011).

The interaction of *scenario* × *within-scenario time* results from a more negative linear decrease of craving in Party compared to Stress, b = -10.62, *SE* = 1.66, *t*(5698) = -6.41, *p* < .001, in Party compared to Café, b = -6.36, *SE* = 1.98, *t*(5700) = -3.22, *p* = .005, in Loneliness/Rumination compared to Stress, b = -7.19, *SE* = 1.63, *t*(5694) = -4.40, *p* < .001, and in Café compared to Stress, b = -4.26, *SE* = 1.72, *t*(5707) = -2.47, *p* = .040. Finally, the main effect *scenario* results from higher craving in Loneliness/Rumination than in Stress, b = 7.87, *SE* = 2.02, *t*(73.2) = 3.90, *p* < .001, in Loneliness/Rumination than in Café, b = 10.06, *SE* = 1.97, *t*(60.7) = 5.11, *p* < .001, in Party than in Stress, b = 5.84, *SE* = 1.91, *t*(75.3) = 3.05, *p* = .009, and in Party than in Café, b = 8.03, *SE* = 1.65, *t*(64.5) = 4.85, *p* < .001.

Loneliness/Rumination and Party (*p* = .222), and Stress and Café (*p* = .141) did not differ significantly.

Comparing the analysis from the main article including all participants, with the above analysis, which included only abstinent participants at the time of VR-CET sessions, reveals mainly similarities but also two differences. 1) While in both analyses the interaction of *presentation* and *scenario* was significant, post hoc tests showed a small difference. The main article analysis indicated that craving during the first presentation was significantly higher compared to the second presentation for all four scenarios, whereas in the supplementary analysis, this effect was only significant for three scenarios and not for Café, where there is only a trend in the same direction. 2) The significant three-way interaction of *presentation* × *group* × *within-scenario time* reported in the main article, was not significant in the supplementary analysis, *F*(1, 5687.6) = 2.30, *p* = .130).

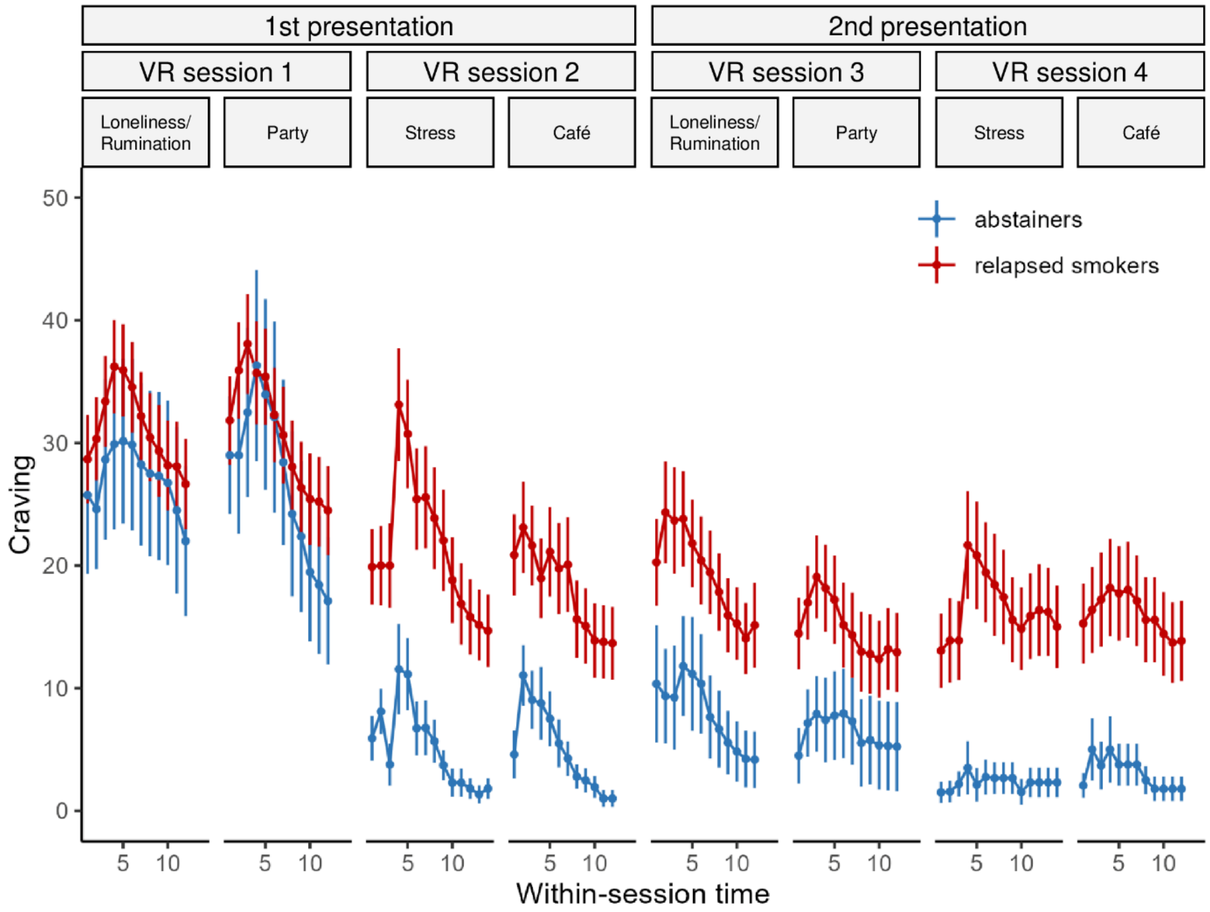

Figure S2. Within and between scenario craving ratings during the course of the VR-CET for future abstainers and relapsed smokers. Only abstinent participants at the time of VR-CET sessions are included. Fifth and tenth craving rating is marked on the x-axis for each scenario. Error bars show standard errors.
